# In Vivo Anti‐Nociceptive Activity of Isoquercetin in Rats Supported by ADMET and Molecular Docking Analysis

**DOI:** 10.1002/cbdv.71490

**Published:** 2026-07-10

**Authors:** Ridha Ben Ali, Dorra Ben Said, Mariam Bourounia, Jalloul Bouajila, Abada Mhamdi, Sihem El Aidli

**Affiliations:** ^1^ Unit of Experimental Medicine, Faculty of Medicine of Tunis University of Tunis El Manar Tunis Tunisia; ^2^ Laboratoiry of Research LR24ES10, Santé Populationnelle Agresseurs Environnementaux Thérapies alternatives Tunis Tunisia; ^3^ Departement of Pharmacology Faculty of Medicine of Tunis University of Tunis El Manar Tunis Tunisia; ^4^ Research Unit n° 17/ES/12 Faculty of Medicine University of Tunis El Manar Tunis Tunisia; ^5^ Laboratoire De Génie Chimique Université De Toulouse Toulouse France

**Keywords:** ADMET analysis, anti‐inflammatory, anti‐nociceptive, antioxidant, isoquercetin, molecular docking

## Abstract

This study aims to evaluate the anti‐nociceptive activity of isoquercetin (IQN) in rats and to determine its mechanism of action using molecular docking. The preliminary study was conducted to determine physicochemical and pharmacological parameters of IQN, an in vitro method was used to assess its antioxidant capacity. Behavioral tests were carried out to evaluate its anti‐nociceptive activity and an in silico molecular docking study was performed to understand the molecule's mechanism of action. The preliminary study demonstrated that IQN is nontoxic to metabolism and organs, with an LD_50_ of 5000 mg/kg, and does not present neuromuscular toxicity. Furthermore, the molecule showed a strong antioxidant capacity. The evaluation of anti‐nociceptive activity in rats revealed a significant anti‐inflammatory effect of IQN. The molecular docking identified a specific action on cyclooxygenases. Thus, IQN appears to be a promising candidate for the treatment of inflammatory pain, supported by its wide safety margin, potent antioxidant.

## Graphical abstract

As a bioactive natural flavonoid, isoquercetin (IQN) emerges as a promising anti‐inflammatory agent. Its anti‐nociceptive activity was substantiated through complementary in vivo experimental models and in silico molecular docking analyses, providing convergent pharmacological and mechanistic support.

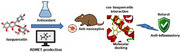

## 1 Introduction

Pain and inflammation are complex biological processes that play a crucial role in host defense but may become deleterious when persistent or dysregulated. Inflammatory pain results from the activation and sensitization of peripheral nociceptors by various mediators released at the site of tissue injury. Among these mediators, prostaglandins are particularly important, as they enhance nociceptor excitability and amplify pain transmission. Prostaglandins are synthesized from arachidonic acid via cyclooxygenase (COX) enzymes, mainly COX‐1 and COX‐2. While COX‐1 is constitutively expressed and contributes to physiological functions, COX‐2 is inducible and primarily responsible for the production of pro‐inflammatory prostaglandins during inflammation. Consequently, inhibition of COX enzymes, especially COX‐2, remains a key therapeutic strategy for managing pain and inflammatory conditions [1, 2, 3].

Natural bioactive compounds, particularly flavonoids, have attracted increasing attention as potential alternatives to conventional anti‐inflammatory drugs due to their multi‐target actions and favorable safety profiles. Quercetin, one of the most abundant dietary flavonoids, is widely present in fruits and vegetables such as apples, onions, tea, and berries. It exhibits a broad spectrum of pharmacological activities, including antioxidant, anti‐inflammatory, antiviral, anticancer, and cardio‐protective effects. These activities are mediated through modulation of oxidative stress, inflammatory signaling pathways, and enzyme inhibition [4, 5, 6].

Quercetin exists in several glycosylated forms that significantly influence its pharmacokinetics, particularly its solubility and intestinal absorption. Among these derivatives, isoquercetin (quercetin‐3‐O‐glucoside, IQN) has gained considerable attention due to its enhanced bioavailability compared to the aglycone form. Recent studies have demonstrated that IQN and structurally related flavonoids exert significant anti‐inflammatory and analgesic effects through multiple mechanisms. These include inhibition of COX‐2 and lipoxygenase (LOX) pathways, suppression of pro‐inflammatory cytokines such as tumor necrosis factor‐alpha (TNF‐α) and interleukin‐6 (IL‐6), and attenuation of oxidative stress [7, 8, 9, 10]. Furthermore, in silico molecular docking studies have suggested that IQN can interact directly with key inflammatory enzymes, including COX‐2, supporting its potential role as a natural inhibitor of prostaglandin synthesis [11, 12, 13].

Despite these promising findings, the pharmacological effects of IQN remain less extensively characterized than those of quercetin, particularly regarding its anti‐nociceptive activity and behavioral outcomes in response to nociceptive stimuli. Most recent studies have focused on its antioxidant and general anti‐inflammatory properties, while limited research has explored its direct effects on pain perception and its precise molecular targets involved in nociception.

Therefore, the present study aims to address this research gap by evaluating the anti‐nociceptive activity of IQN in an experimental rat model and elucidating its mechanism of action using molecular docking approaches. Particular emphasis is placed on its interaction with cyclooxygenase enzymes, which are central to prostaglandin‐mediated pain and inflammation. This combined in vivo and in silico investigation may contribute to the development of IQN as a promising natural therapeutic agent for the management of pain and inflammatory disorders.

## 2 Results and Discussion

### 2.1 Preliminary Study

#### 2.1.1 ADMET Prediction

The solubility(S) of IQN in water was determined in silico by *SwissADME* according to the method described by Ali et al., in 2012 with a log(S) = ‐4.35 which means moderate solubility of the molecule in water [14].

The toxicity of IQN was assessed in silico by *ProTox 3.0*. The found results showed that the molecule studied in this work was classified among the less toxic molecules, with a high lethal dose 50 (LD_50_) of 5000 mg/kg.

The toxicity of IQN on organs and on cytochromes was also predicted by *ProTox 3.0*. The found results showed that the molecule has respiratory and renal toxicity with significant probabilities and has no toxicity on the liver, the nervous system, the heart and the cytochromes. IQN is much less toxic than quercetin which has a lethal dose (LD_50_) of 159 mg/kg [15, 16].

#### 2.1.2 In Vitro Evaluation of Antioxidant Capacity of IQN

**FIGURE 1:**
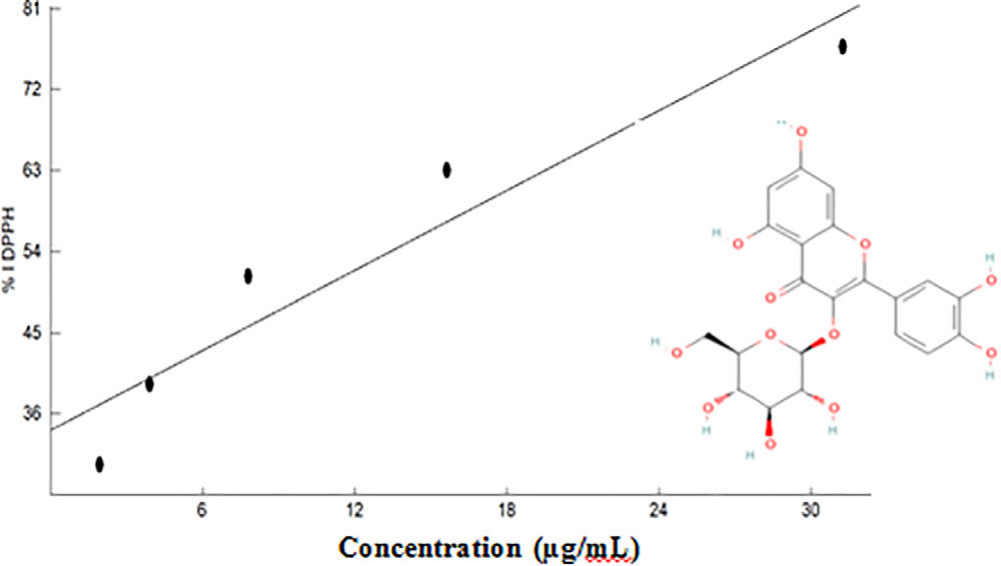
Percentage of DPPH inhibition as a function of IQN concentrations. (Correlation *r* = 0.95, EC_50_ = 10.7 µg/mL)

The found results of diphenyl‐1‐picrylhydrazyl (DPPH) test has showed that the IQN molecule has important antioxidant activity proven by a low effective concentration (EC_50_ = 10 µg/mL). (Figure 1).

These results are in accord with those of a previous study who evaluated the antioxidant activity of quercetin [17]. Thus, the antioxidant capacity of IQN is higher than that of quercetin. Indeed, the glucose group present in IQN could be responsible to ameliorate its antioxidant activity [18].

This suggests that a molecule with important antioxidant capacity can be used to limit the deleterious effects of oxidative stress. Oxidative stress and inflammation frequently interact and cause aggravation disease which interacts with inflammation and frequently causes disease aggravation. In this case, an anti‐inflammatory molecule which has significant antioxidant activity may be very interesting as a treatment to develop. To do this, we tested the anti‐inflammatory activity of IQN in rats.

#### 2.1.3 In Vivo Verification of the Effectiveness of IQN per os Administration

In order to verify the effectiveness of the oral administration route of IQN in rats, high‐performance liquid chromatography (HPLC) assay was used.

The concentration of IQN present in the different samples was calculated using the calibration curve (Table 1).

**TABLE 1:** IQN levels in the different samples after HPLC analysis in sample extraction of plasma and organ rats.

Samples	Plasma	Urine	Liver	Kidney	Brain	Heart	Intestine
**[IQN] µg/mL**	35.07	58.18	22.96	20.49	0	0	0

The analysis of the results obtained shows the presence of IQN in plasma, urine, liver, and kidney, and these results are in accord with the bibliography [19]. These results confirm the effectiveness of the per os route which will be chosen in the treatment of rats by IQN to evaluate its biological activity.

In 2020, Maharni et al. [15] showed, using prediction tools, that IQN does not cross the blood–brain barrier, which explains the absence of this molecule in the brain.

### 2.2 In Vivo Evaluation of Biological Activity of IQN in Rats

#### 2.2.1 Evaluation of Anti‐Nociceptive Effect of IQN

This test consists in inducing inflammatory abdominal pain in rats by intra‐peritoneal injection of acetic acid. One hour before the injection of acetic acid, the rats are per os treated with IQN at different doses. Thus, the evaluation of the anti‐nociceptive effect of this molecule is done by comparing the number of writhing to control group no treated with IQN.

According to the bibliography, in the case of nociception induced by a chemical stimulus such as formalin injection there is an immediate early phase of intense pain, which is followed by a late phase of moderate pain [20].

In this work, following the nociception induction, the early phase began 10 min after the injection of acetic acid and extended over 30 min. So, the late phase occurred about 10 min after the end of the first phase and lasted 10 min.

**FIGURE 2:**
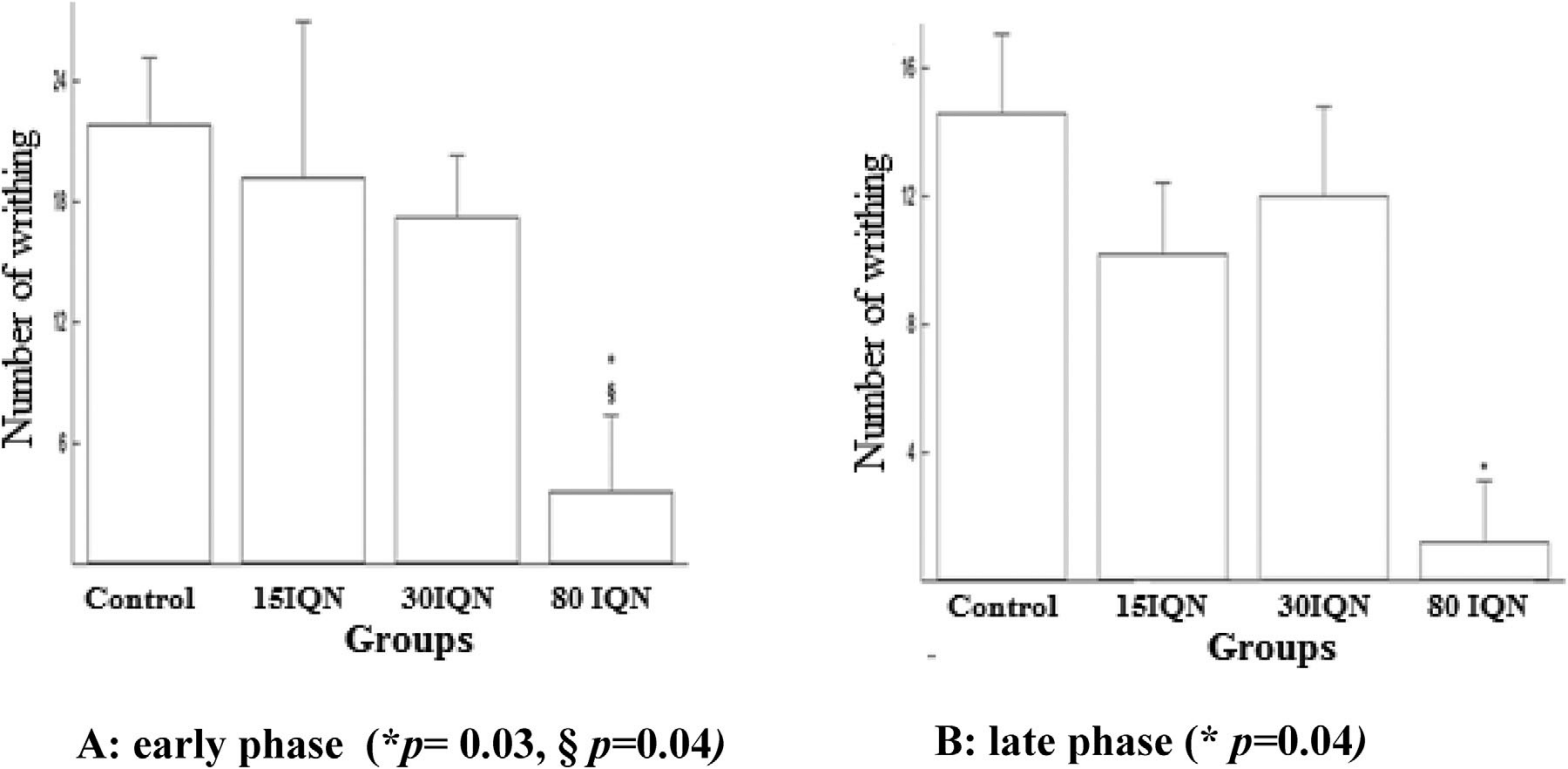
Effect of IQN on the number of writhing during the (A) early (B) and late phases after inflammation induction. (Mann–Whitney *U* test (*n* = 5), *SD*: *p < 0.05 versus control group, §*p <* 0.05 vs. 30INQ group).

The results found during the two phases of inflammation (Figure 2) showed that the number of writhing observed in rats insignificantly decreased under the effect of IQN treatment at doses of 15 and 30 mg/Kg. However, the dose of 80 mg/kg showed a significant reduction in the number of writhing compared to the control group and compared to the group treated with the dose of 30 mg/kg of IQN (Figure 2).

Oxidative stress plays a central role in the pathophysiology of inflammation and pain through the overproduction of reactive oxygen species (ROS), which activate pro‐inflammatory signaling pathways. IQN, as a glycosylated derivative of quercetin, exhibits strong radical‐scavenging activity due to its hydroxyl groups, thereby reducing ROS levels. This antioxidant effect may attenuate lipid peroxidation, protein oxidation, and neuronal sensitization. Furthermore, the anti‐nociceptive effect of IQN may be due to its anti‐inflammatory activity or to its analgesic capacity. In this case, to check its action we used the hot plate test.

#### 2.2.2 Evaluation of Analgesic Activity of IQN

Based on the bibliography, we find that quercetin‐3‐O‐β‐glucopyranoside has analgesic activity which appears 60 min after treatment at a dose of 50 mg/kg [21]. For this, we chose to work with IQN at increasing doses of 5, 15, 30, and 80 mg/kg, dissolved in water and administered to the rats orally one hour before the exposure to thermal stimulus (46°C), measuring the latency time.

**FIGURE 3:**
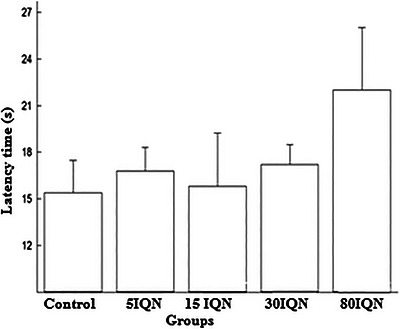
Evaluation of analgesic effect of IQN in rats by hot plate test measuring the latency time (Mann–Whitney *U* test (*n* = 5), *VS* control group *p (0.3–0.8)*>0.05).

The results found (Figure 3) showed that the treatment of rats with IQN caused a nonsignificant increase in latency time following exposure to the thermal stimulus. Indeed, IQN does not have analgesic activity. Following these results, the anti‐nociceptive activity of IQN shown in the acetic acid test is most likely due to its anti‐inflammatory activity.

The present findings are consistent with previous studies on IQN and structurally related flavonoids such as quercetin, rutin, and kaempferol. Quercetin has been widely reported to exert anti‐inflammatory and analgesic effects through COX inhibition, suppression of pro‐inflammatory cytokines, and antioxidant mechanisms [8].

#### 2.2.3 Evaluation of Neuromuscular Toxicity of IQN Using Behavior Test

In order to verify the neuromuscular toxicity of the IQN molecule, we used the hanging suspended string test. This test consists to in measuring the rat's time fixation and its ability to wrap itself on the string.

**FIGURE 4:**
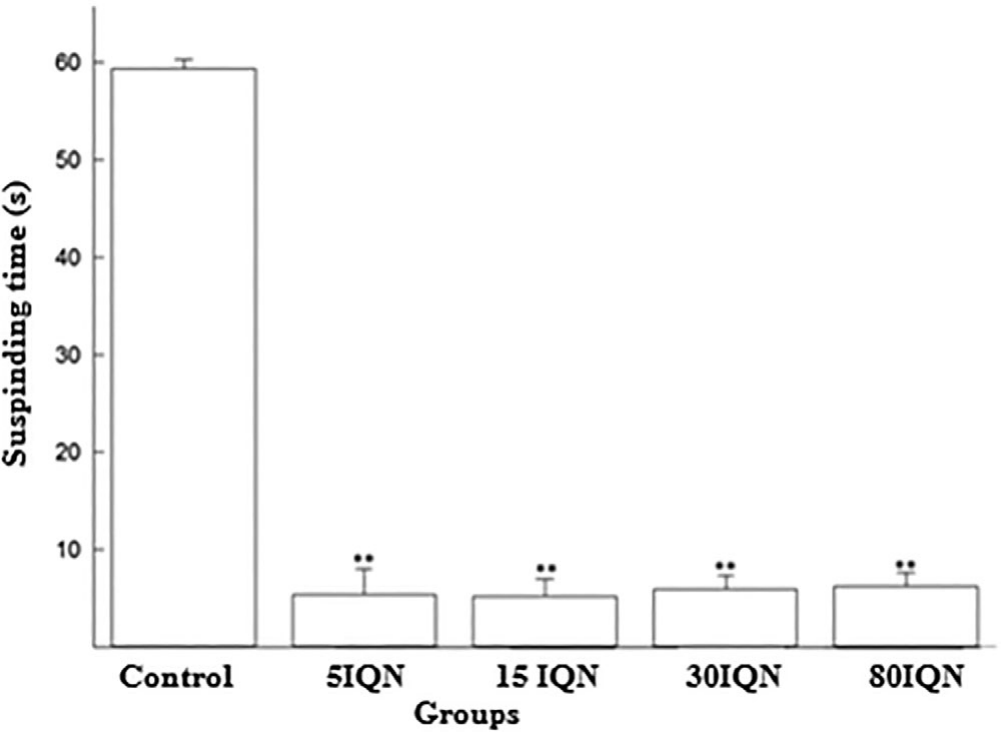
Effect of IQN on fixation time in suspended string (Mann–Whitney *U* test (*n* = 5), **p < 0,001 vs. control group).

The found results showed that control rats suspended by their forelegs for 1 min, while rats treated with IQN at increasing doses from 5 mg/kg to 80 mg/kg quickly freed themselves from the wire quickly after only a few seconds (5 s with *p* < 0.001) (Figure 4). The reduction in the fixation time of rats on the suspended string under the effect of IQN does not depend on the dose administered.

The reaction of treated rats which suspended for a short time with tail wrapping around the string may be due to the presence of an anxiolytic and relaxing effect of IQN than to the presence of neuromuscular toxicity. In order to check the state of anxiety in rats, the elevated maze test was used in evaluation of anxious responses. In order to verify that the results obtained in the hanging wire test are associated or not with behavioral changes linked to anxiety, the rats were subjected to the elevated cross maze test, this test is recognized for evaluating anxious responses.

**FIGURE 5:**
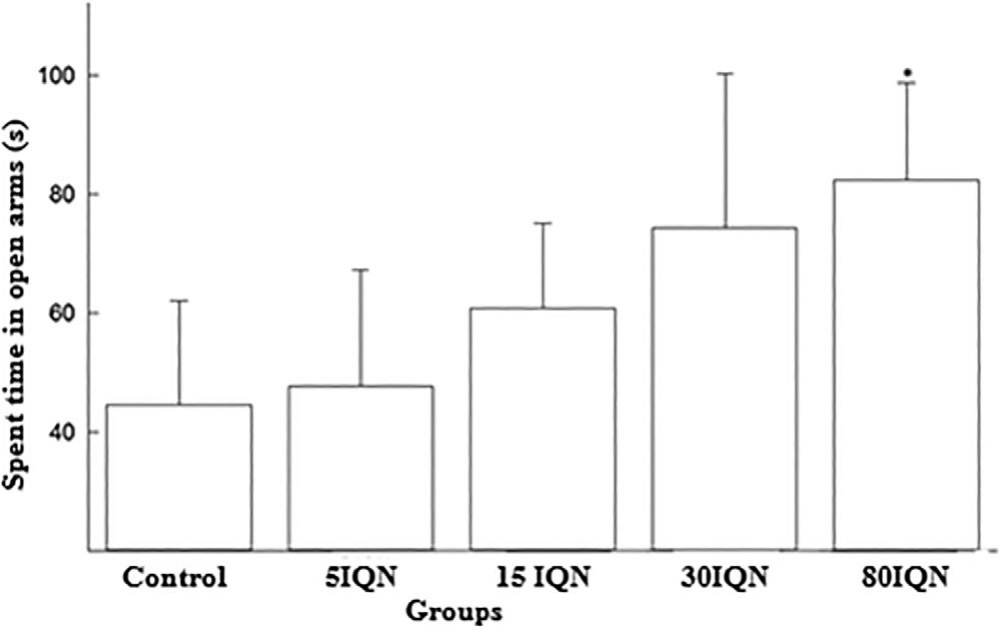
Effect of IQN on spent time in open arms of elevated plus maze (Mann–Whitney *U* test, (*n* = 5), *p = 0.05 vs. control).

The found results showed an increase in the time spent by the rats in the open arms under the effect of IQN treatment. Thus, the observed effect was dose‐dependent with a maximum obtained following treatment with a dose of 80 mg/kg (Figure 5).

Previous studies [22] suggest that quercetin has an anxiolytic effect [23, 24]. In our study, exploratory behavior was observed in treated rats, manifested by more frequent movements and increased exploration of the maze. This observation suggests that the rats overcame their fear under the effect of IQN. In this case, the duration of fixation of rats treated on the suspended string test cannot be explained by the anxiety of the animal under the effect of IQN which is anxiolytic, but can be explained by the ability of the animal to overcome fear and face danger by avoiding the state of phobia. Based on the bibliography we find that caffeine increases the state of vigilance and reduces phobia [25].

### 2.3 In Silico Determination of IQN Mechanism Target by Molecular Docking

Molecular docking and in vitro studies have demonstrated that quercetin derivatives can bind to the active site of COX enzymes, stabilizing interactions through hydrogen bonding and hydrophobic contacts, thereby reducing enzymatic activity [3].

In this work, we showed that IQN has anti‐inflammatory activity and in order to determine its mechanism target we used an in‐silico study by testing the interaction of IQN with the proteins involved in the inflammatory process; cyclooxygenase (COX‐1), phospholipase (PLA2) and histaminic receptor (RH4).

**FIGURE 6:**
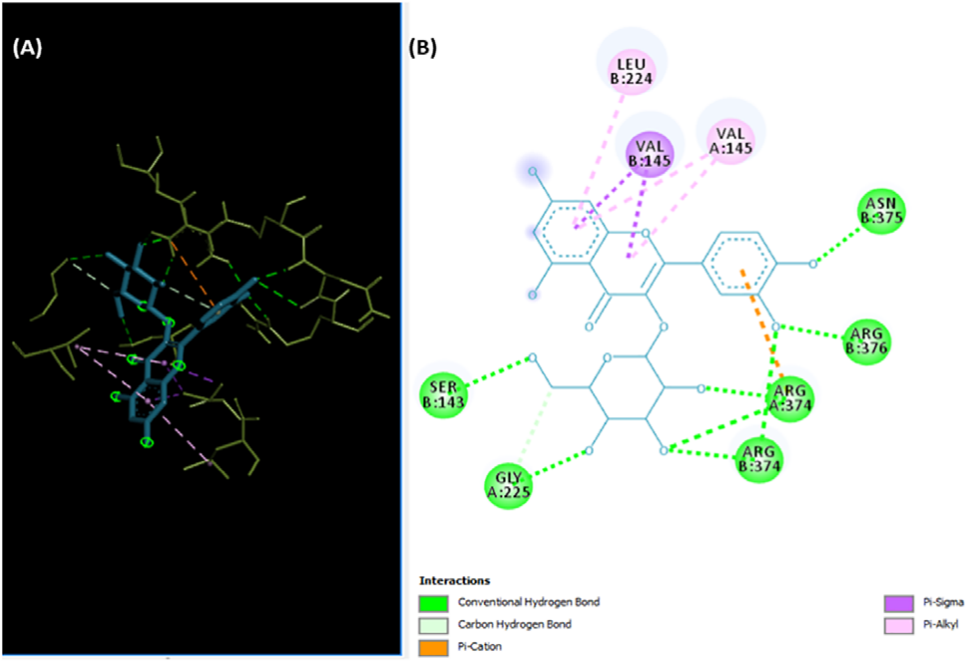
Visualization of IQN interactions with COX‐1: (A) 3D Structure of COX‐1‐IQN complex; (B) structure 2D of COX‐1‐IQN complex.

**FIGURE 7:**
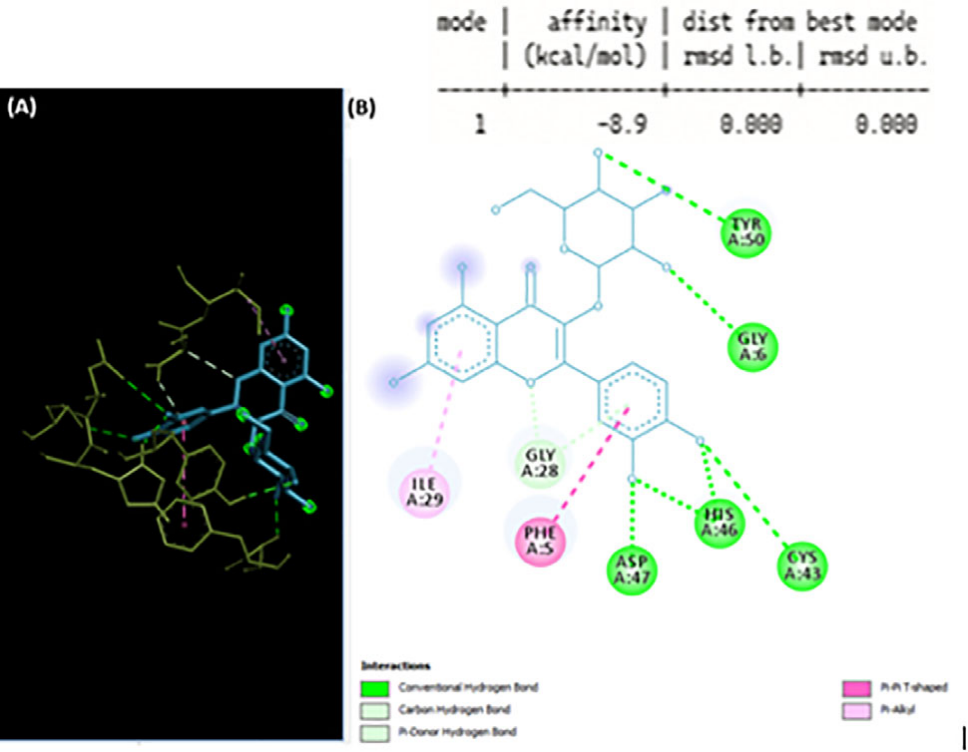
Visualization of IQN interactions with PLA2: (A) 3D Structure of PLA2‐IQN complex; (B) 2D Structure of PLA2‐IQN interactions.

**FIGURE 8:**
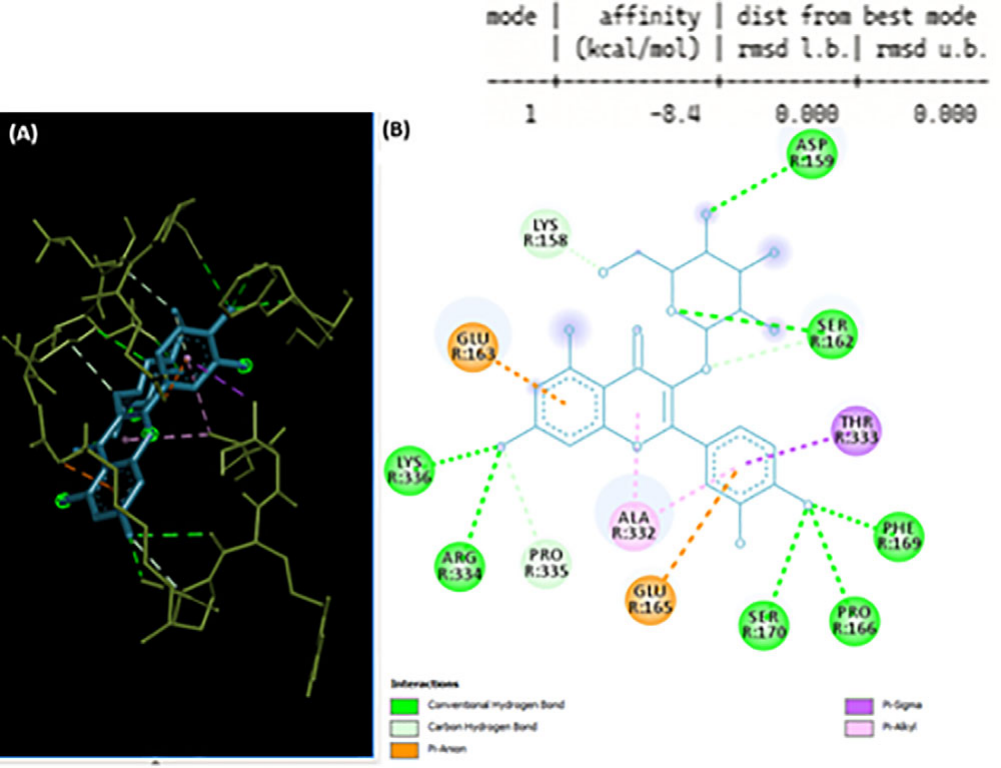
Visualization of IQN interactions with RH4: 3D Structure of RH4‐IQN interactions; (B) 2D Structure of RH4‐IQN interactions.

The obtained results (Figures 6, 7, 8) showed that the best interaction scores of each receptor–ligand complex for COX‐1, PLA2 and RH4 with IQN are respectively ‐10.9, ‐8.9, and ‐8.4 kcal/mol. In comparison with quercetin which showed affinities of ‐9.4 kcal/mol for COX‐1 [26] and ‐7.06 kcal/mol for PLA2 [27], IQN showed important affinities to these proteins. However, it kept the same affinity as quercetin for RH4 with a score of ‐8.7 kcal/mol [28].

The interaction of IQN with residues ASN375, ARG376, ARG374, and SER143 of COX‐1 (Figure 6) involves only conventional hydrogen bonds that are important for stabilizing the protein–ligand complex.

The interaction with GLY225 involves both a conventional hydrogen bond and a carbon‐hydrogen bond which are considered weaker hydrogen bonds when the donor is a polarized carbon [29]. It also forms two hydrophobic Pi‐Alkyl‐type bonds with the amino acids VAL145 and LEU224 that occur when an aromatic ring interacts with a nearby alkyl group, and a single Pi‐Sigma‐type bond with VAL145 [29].

The interaction of IQN with residues TYR50, GLY6, ASP47, CYS43, and HIS46 of PLA2 (Figure 7) involves conventional hydrogen bonds. The interaction with GLY28 involves both a “Pi‐Donor” hydrogen bond and a carbon‐hydrogen bond. It also forms a Pi‐Alkyl‐type bond with ILE29 and a “Pi–Pi T‐shaped” bond with PHE5, which occurs when two π ring systems are located relative to each other in a T‐shaped arrangement [29].

The interaction of IQN with residues ASP159, SER162, PHE169, PRO166, SER170, ARG334, and LYS336 of RH4 (Figure 8) involves conventional hydrogen bonds. The interaction with ALA332 involves two ‘Pi‐Alkyl’ type bonds. It also forms two carbon–hydrogen bonds with PRO335 and LYS158, two “Pi‐Anion” type bonds with GLU163 and GLU165, which exists between a negatively charged atom and the electrons of a delocalized π system [29, 30] and a Pi‐Sigma bond with THR333.

Thus, the molecular docking results revealed important binding affinity of IQN toward COX‐1, PLA2, and RH4, three targets involved in inflammatory processes [31]. The predicted ligand–receptor complexes were characterized by several stabilizing interactions and the absence of unfavorable contacts, suggesting stable binding within the active sites of these proteins. Such interactions may interfere with the biological activity of COX‐1 and PLA2, leading to a reduction in the production of pro‐inflammatory mediators derived from arachidonic acid metabolism. In addition, the interaction of IQN with RH4 may contribute to the modulation of inflammation‐related signaling pathways.

Collectively, these findings provide a plausible molecular basis for the anti‐inflammatory activity of IQN observed in the experimental studies.

## 3 Conclusions

Overall, the anti‐nociceptive activity of IQN can be attributed to a multifactorial mechanism involving inhibition of COX‐mediated prostaglandin synthesis, reduction of oxidative stress and ROS‐mediated signaling, and modulation of pain pathways through antioxidant‐dependent and independent mechanisms. These findings are in agreement with the growing body of literature on flavonoids and support the therapeutic potential of IQN as a natural analgesic and anti‐inflammatory compound.

## 4 Experimental Section

The isoquercetin (**quercetin 3‐O‐beta‐D‐Glucoside)** (IQN) (Figure 1) used in this study was purchased in the form of a yellow powder and supplied as part of collaboration with the Chemical Engineering Laboratory of University of Toulouse III.

### 4.1 Preliminary Study

#### 4.1.1 ADMET Prediction

Solubility and certain pharmacokinetic parameters of IQN were determined from the chemical formula using online computer analysis by *SwissADME* (http://www.Swissadme.ch/). Furthermore, the prediction of the toxicity of the molecule was carried out by online modeling using the address (https://comptox.charite.de/protox3/).

#### 4.1.2 Monitoring of IQN Level in Rat Organism

In order to verify the stability of the IQN and the effectiveness of the oral route administration chosen in this study, we developed a high‐performance liquid chromatography (HPLC) monitoring method.

The IQN monitoring was carried out in a rat treated 3 days before sacrifice with a dose of 50 mg/kg of IQN orally. The treated rat was sacrificed 1 h after treatment on the third day. Blood, urine and organs (heart, kidneys, liver, intestine, and brain) were collected, homogenized in saline solution and stored at −20°C.

➢ ***HPLC Conditions***

The used HPLC methodology was equipped with a manual injector, an oven (25°C–90°C), an injection pump and a UV/Vis detector. This device is linked to control and integration software (ChemStation‐Agilent Technologies).

Based on the bibliography and after optimization, the determination of IQN levels by HPLC was carried out under the following conditions [11]:

• Column: C18 (4.6×250 mm id), • Temperature: 50°C, • Flow rate: 1 mL/min,• Wavelength: 375 nm, • Mobile phase: Acetonitrile/bi‐distilled water (70:30 v/v), in isocratic mode.

➢ ***Isoquercetin Extraction***

For the quantification of the IQN by HPLC in a biological matrix, we opted for a liquid/liquid extraction as in the studies cited in the literature [32, 33]. The extraction procedure is detailed in Table 2.

**TABLE 2:** Procedure of Isoquercetin extraction from plama sample or organ homogente obtaned from tratred rat with IQN at 50 mg/kg dose per os.

Tubes	Tube 1 Control (0 IQN):	Tubes 2 to 6 calibration curve	Tubes 7 to 13 Sample of treated Rat with IQN:
**Plasma [IQN] = 0**	+ 200 µL	+150 µL	—
**Isoquercetin solution**	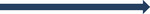	+50 µL of IQN solution at increasing concentrations at each tube: 50/ 100/ 150/ 200/ 250 µg/mL	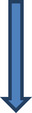
**Plasma or Urine or** **Homogenatetissue**	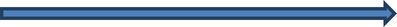		+200 µL
	Each tube 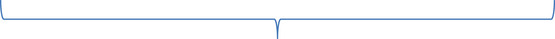		
**Acetonitrile**	+200 µL		
+Vortex for 1 mn			
+Centrifugation for 20 min at 4000 rpm at 4°C			
+Injection of 50 µL of the upper phase into the HPLC system			

#### 4.1.3 Diphenyl‐1‐picrylhydrazyl (DPPH) Radical Scavenging Assay

The DPPH assay was based on the method reported by Blois [34].

IQN sample was added at different concentrations (0,97–15,6 µg/mL) to 1 mL of 1,1‐diphenyl‐2‐picrylhydrazyl (DPPH) dissolved in methanol to 100 mM. The mixture was incubated at room temperature for 30 min and then the absorbance of stable DPPH was determined at 517 nm using a UV/vis spectrophotometer. The concentration of DPPH was calculated using a standard curve. The free radical scavenging activity was expressed as follows:

$$\%\mathrm{Inhibition}=\left(\left(C_{0}-C_{sample}\right)/C_{0}\right)*100$$

where, *C*_0_ = Concentration of DPPH in negative control and *C*_sample_ = Concentration of DPPH in the sample.

### 4.2 In Vivo Study of the Antalgic Activity of IQN

#### 4.2.1 Animals

Six‐week‐old healthy male *Wistar* rats (100 rats) were housed by pairs in cages (25/50 cm) and maintained at 23°C, 12/12‐h light/dark cycle under specific pathogen‐free conditions. The rats were allowed to acclimatize in the experimental medicine unit for a period of one week before the beginning of the study. During the experiment period, they received a commercial pellet diet and water ad libitum. Rats weighing 200 g were used for the experiments. All experimental procedures were approved by the Ethics Committee of the School of Medicine of Tunis according to the standards of the International Council for Laboratory Animal Science (ICLAS). This committee does not issue formal ethical approval numbers for animal studies. Therefore, no approval number is available.

#### 4.2.2 Choice of Treatment Dose With IQN

Based on the literature, quercetin‐3‐O‐β‐glucopyranoside has been found to have analgesic activity that manifests 60 min after treatment at a dose of 50 mg/kg [21]. Another study found that quercetin‐3,7‐O‐dimethyl ether has an analgesic effect at lower doses (3, 6, and 9 mg/kg) [35]. Therefore, the treatment doses with IQN in this work were used at increasing doses of 5, 15, 30, and 80 mg/kg.

#### 4.2.3 Experimental Animal Model

The rats were divided into five groups (*n* = 5), four of which were treated with 5 mL/kg of increasing doses of IQN by oral gavage (5, 15, 30, and 80 mg/kg per os) and the control group received normal saline. One hour after treatment, behavioral tests were carried out in rats.

➢ ***Writhing test***

Each rat treated per os by IQN, was hurt 30 min later by an intraperitoneal injection of a 3% acetic acid aqueous solution (5 mL/ kg body weight). The rat was immediately placed in a transparent observation cage and the number of writhing was counted for 60 min.

➢ ***Hot Plate test***

The hot plate test is a behavioral model of nociception which is commonly employed to screen analgesic drug effects. During a hot plate test, rats display several noxious‐evoked patterns as well as exploratory and self‐care responses. The Hot plate test uses the reflexes of the thermal pain due to the contact of the paws with a heated surface. The rats were placed on a heated plate maintained at a constant temperature of 48°C. During the hot plate test period, we noted the latency time before the rat elevated and licked its paws and jumped along [36].

➢ ***Elevated plus maze***

Anxiety was assessed using the elevated plus maze. The rat was given the choice of spending time in open and unprotected maze arms or enclosed and protected arms; the maze was approximately 60 cm from the floor [37, 38]. The closed arms represent security, while the open arms test the exploratory capacity of rats. The session duration was 2 min and began when the rat was placed in the center of the maze facing an open arm. During this experiment the following parameters were measured: time spent in the open arms and time spent in the closed arms. This test was repeated three times successively with a 5 min stop each time. The elevated maze apparatus was wiped out using a 10% alcohol solution before the following animal was introduced, so as to preclude the possible cuing effects of odors left by previous subjects.

➢ ***Suspended string***

The suspended string test was designed to evaluate the muscular strength of the animals. The rats were hung by their two forepaws from the middle of a wire (50 cm in length and 1 mm in diameter) and the holding time was measured, with a maximal time of 60 s. Each animal was submitted to three trials separated by 15 min intervals, during which it was returned to its home cage [38].

#### 4.2.4 Statistical Analyses

The results stated as averages ± standard deviation. All analyses were carried out with *Biostat* software for Windows. Significant differences between rats groups (*n* = 5) were determined by the Mann–Whitney *U* test (nonparametric test) for multiple comparisons with statistical significance when *p* < 0.05.

### 4.3 Molecular Docking

The ligand molecule (quercetin‐3‐o‐glucoside) was drawn using the freeware *ChemSketch* software and saved using *Discovery Studio* in “.pdb” format.

The structures of the protein molecules (phospholipase A2, cyclooxygenase 1, and histamine H4 receptor) were downloaded from the Protein Data Bank website (*rcsb.org*), respectively with the following identifiers: 3N8V, 6KQU, and 8HN8.

The downloaded protein structure is edited either by the *Swiss Pdb Viewer* (*SPDBV)* application or by *WordPad*.

The interaction region was identified from the website (https://prankweb.cz/).

Molecular Docking analysis was generated using the *Auto Dock‐Vina tool*.

The positions found were saved by the *Python Molecular Viewer (PMV)* software.

The 2D and 3D diagrams of the ligand‐receptor interactions in each position were visualized using *Discovery Studio* software.

## Author Contributions

**Ridha Ben Ali**: conceptualization, software, supervision, methodology, validation, formal analysis, visualization, project administration, writing – original draft. **Dorra Ben said**: investigation, writing – review editing. **Mariam Bourounia**: data curation, investigation, writing – review editing. **Jalloul Bouajila**: writing – review editing, resources. **Abada Mhamdi**: resources. **Sihem El Aidli**: resources.

## Conflicts of Interest

The authors declare no conflicts of interest.

## Data Availability

The data that support the findings of this study are available from the corresponding author upon reasonable request.
